# For feminist geographies of austerity

**DOI:** 10.1177/03091325211065118

**Published:** 2022-01-14

**Authors:** Sarah Marie Hall

**Affiliations:** School of Environment, Education & Development 409567 , The University of Manchester, Manchester, UK

**Keywords:** Austerity, feminist, social reproduction, epistemology, intersectionality, silence, embodied fieldwork

## Abstract

Austerity policies and austere socio-economic conditions in the UK have had acute consequences for everyday life and, interconnectedly, the political and structural regimes that impact upon the lives of women and marginalised groups. Feminist geographies have arguably been enlivened and reinvigorated by critical engagements with austerity, bringing to light everyday experiences, structural inequalities and multi-scalar socio-economic relations. With this paper I propose five areas of intervention for further research in this field: social reproduction, everyday epistemologies, intersectionality, voice and silence, and embodied fieldwork. To conclude, I argue for continuing feminist critique and analyses given the legacies and futures of austerity.

## I Introduction

Austerity is a profoundly feminist issue. A rich and growing body of feminist geographical research has argued that austerity, as a political choice rather than inevitability, is a multi-scalar, temporal and spatially unjust process and experience (Greer Murphy, 2017; Hall, 2019a, Stenning, 2018). Austerity policies and austere socio-economic conditions are known to have consequences for both everyday life and, interconnectedly, the political and structural regimes which impact upon the lives of women and marginalised groups (Craddock, 2017; Hall, 2019b; Pearson, 2019). Austerity serves to accentuate these inequalities at multiple scales, from family, household and community relations to sectors of the economy in which women are the main recipients and providers (Davies and O’Callaghan, 2014; Hall, 2020a; MacLeavy, 2011).

Austerity in the UK has attracted a burgeoning array of geographical scholarship. This could be interpreted as a representation of the strength of feeling towards the austerity agenda so diligently pursued by the UK government since 2010, following the 2008–2010 Global Financial Crisis. The government posited austerity as being for the public and national good, a necessity to ensure a stable economic future less reliant on foreign borrowing and a trimmer, more streamline state infrastructure resilient to global crises. Changes in the name of austerity have been widespread, acute and devastating. They have compounded and exacerbated longer-term trends with the dismantling of the welfare state, the neoliberal roll-back of government and the increasing responsibilities of individuals for their economic condition (Harrison, 2013; Horton, 2016; Horton et al., 2021; Pimlott-Wilson, 2017). Geographers have paid particular interest in challenging the discourses, policies, practices, politics and imaginaries underlying austerity in the UK, embedded in these longer genealogies (Horton, 2016; Werner et al., 2017). It is also probable that the dominance of English-speaking geographies has propelled the flourishing of work on austerity in this context, as well as in the US (see Fregonese, 2017; Müller, 2021). This paper uses this body of work as a scholarly mooring, whilst acknowledging that austerity is socially and spatially differentiated, emplaced and embraced in different ways in different places and times. Despite a focus on UK-based literatures, throughout this review and where space permits, I also gesture to global literatures on feminist geographies of austerity.

Feminist geographies are herein defined according to the authors’ self-identification, works referenced, or conceptual framings used – including work on gender, family, care, intimacy and social difference. This is tempered by an understanding that feminist geographies extend and draw together scholarship across critical race, post-colonial, post-human, queer and women’s studies. Indeed, recent years have seen a flourishing of heterodox economic geographies (Bonds, 2013; Macleavy et al., 2016; Werner et al., 2017), which draw upon and contribute to multifarious and diversely interconnected subdisciplinary debates, including health, demography, critical GIS studies and more.

Revealing the deep and painful impact of austerity cuts through detailed theoretical reworkings, ethically considered empirical engagements and politically charged praxis, feminist geographies have influenced the form and tone of geographical debates in this field. More than this, feminist geographical approaches, critiques and contentions have been reasserted and enlivened through this focus on austerity, particularly the field of feminist political economy (MacLeavy et al., 2016; Montgomerie and Tepe-Belfrage, 2016; Werner et al., 2017). Within these accounts, everyday experiences, structural inequalities and multi-scalar socio-economic relations are highlighted. By bringing geographical attentions back to inequality, care, social relationships, social reproduction, welfare, and poverty and disadvantage, this work demonstrates the timeliness of critical feminist perspectives.

This paper is written with an agenda to synthesise where feminist geographical analyses of austerity have been, where they might go next and what political and economic issues are at stake with this work. To do this, I first chart out contributions made within broader feminist studies regarding austerity, including feminist economics, politics and sociology, and then identify how feminist geographies have shaped these discussions, with a particular focus on the UK context. Building on these rich foundations, with the rest of the paper I outline five areas with scope for further feminist geographical contributions regarding austerity: social reproduction, everyday epistemologies, intersectionality, voice and silence, and embodied fieldwork. Within this, I show how each theme further builds upon feminist contributions around everyday experiences, structural inequalities and multi-scalar socio-economic relations. My aim, then, is to advance debates within feminist geographies of austerity, by identifying directions for propelling forward these collective discussions. To close, I identify the necessity in continuing with these critiques, given the legacies of austerity and the likely impacts for generations to come.

## II Feminism and austerity

Feminist activists have been amongst the most vocal critics of the divisive and discriminatory austerity measures introduced in the UK since 2010 (Bassel and Emejulu, 2017; Craddock, 2017; Davies and O'Callaghan, 2014). Charities, think-tanks and lobbying groups such as the Fawcett Society, Focus E15, Runnymede, Sisters Uncut, UK Feminista and Women’s Budget Group have launched powerful critiques of cuts to public expenditure in health care, welfare, disability support, education, social and childcare, and social housing – and how these policies disproportionately impact on women (Pearson, 2019). Prominent in public discussions, they have shaped debates about austerity politics, encouraging resistance through campaigns and publications (e.g. Fawcett Commission, 2012; Sisters of Frida, 2012; Sisters Uncut, 2019; WBG, 2018).

It would, however, be a false dichotomy to separate feminist activism from feminist academia, given their sustained dialogue and interconnections (Federici, 2012; Hall, 2020a; Hanisch, 1970; Pearson and Elson, 2015). This is particularly the case for expertise within feminist economics. Underpinned by the notion of gender-inclusive economics, feminist economics encompasses the models, methods and modes of analysis for interpreting and developing economic systems, processes and measures (Ferber and Nelson, 1993). Scholars in this field have played an important role in highlighting the uneven assumptions and outcomes of austerity policies, going ‘beyond chronicling the impact on women of policies that privilege markets and money over people’ to provide ‘an understanding of the economy as a gendered structure’ (Pearson, 2019: 29; Pearson and Elson, 2015; Perrons, 2015).

A key contribution of this work is the conceptualisation of the household as a social and political economy unto itself, emplaced within wider labour markets and state policies (Elson, 1998; Folbre, 1986). With this approach, developed in the context of concerns around the gendered impacts of recession and neoliberalism in the UK in the 1980s, responsibilities for reproductive labour are understood alongside changing experiences of employment and the labour market (Bradley, 1986; Rubery and Tarling, 1988; Walby, 1986). The shift in women’s participation in service economies and flexible, part-time working also raised questions about reproductive labour, as seen in earlier international campaigns of Wages for Housework (Federici, 2012). This scholarship and activism highlighted how women are often responsible for managing everyday household budgets and are, therefore, more likely to bear the ‘brunt’ of economic crises (Volger 1994: 248).

The household remains a key feature in feminist writings on the everyday impact of crises, including for projecting the scale of impacts at national and regional levels (Chant, 1997; Roberts, 2016). It is posited that ‘looking [at austerity] through a feminist lens casts new light into the black-box of household […] to make visible the experimental nature of economy storytelling’ (Montgomerie, 2016: 419–420). The impacts of austerity across Europe have also been described as ‘entanglements between state-directed coercion and the household’ (Bruff and Wohl 2016: 92). The household is also referred to ‘as a lens for the analysis of austerity’ in the UK, a ‘key device in austerity politics’ (Tepe-Belfrage and Wallin, 2016: 390–391).

Within both mainstream and feminist economics, ‘households’ are normalised as the scale at which to observe change, a category for socio-spatial comparisons (Himmelweit et al., 2013; Volger, 1994). The household is used as the proxy for everyday practices, spaces and relationships (including family and domestic activity), a unit of analysis to measure the socio-economic impacts of austerity cuts. However, feminist political and geographical research has been critical of this approach, arguing that while drawing attention to structural inequalities, it can obscure everyday experiences and socio-economic relations that occur *within* and *between* households (Hall, 2016). A focus on households can also have the effect of writing-out issues of intimacy, care and family that do not neatly fit this scale of analysis (Hall, 2016).

Further interdisciplinary feminist scholarship has also highlighted the damaging consequences of public cutbacks to already underfunded public resources for minority women and vulnerable communities in the UK. This work reveals the intimate intersections of gender, race and migration within the politics and lived experiences of austerity (Bassel and Emejulu, 2017; Erel, 2018). Work on ‘austerity parenting’ (Jensen and Tyler, 2012; Jensen, 2018) has been crucial in highlighting the inherent tension in parents being held responsible for their children’s presents and futures in the context of an increasingly retreating and irresponsible state. Contributions on the ‘politics of austerity’ also emphasise responses to and discourses of feminism, austerity and crisis: that ‘whether in recovery or crisis, neoliberal economics and politics have proved deeply destructive to most women, and have exacerbated the intersecting divides of gender, race, ethnicity, sexuality and class’ (Brah et al., 2015: 7; Craddock, 2017; Pearson and Elson, 2015). These contributions have also been formative for feminist geographies of austerity.

## III Feminist geographies and austerity

Feminist geographical research has a broad ethos and set of motivations, anchored by concerns about, and attempts to address, oppression caused by difference, power and inequality (Silvey, 2012). With key interests in scale, subjectivity, power, politics, difference and diversity (McDowell, 1993), feminist geographies represent a unique and much-needed interjection in academic debates about the impacts of austerity. Namely, a confluence of writing within feminist geography has interrogated austerity politics, policies and experiences, probing at the meanings, makings and motivations of austerity. The everyday impacts of state cuts have drawn much attention, particularly intersecting social inequalities across space. The premise here is that the social and gendered (and race, class and disability related, and more) impacts of austerity ‘are not evenly dispersed’ (Greer-Murphy, 2017: 2), deepening already-existing uneven socio-economic relations.

Work on youth experiences of austerity, for instance notes the gendered and classed bodywork entailed in service work, as an industry that witnessed increasing competitiveness for jobs in the context of rising unemployment and welfare cuts (McDowell, 2012, 2017). Additionally, it has been demonstrated that everyday youth aspirations and futures have been reconfigured in the face of deep austerity cuts (Horton, 2016; Pimlott-Wilson, 2017). Life-courses, lived and imagined, can be profoundly impacted by cuts to welfare, public services and childcare, unemployment, unattainable rents and austerity has also shaped gendered and intergenerational care-giving and receiving (Jupp et al., 2019; MacLeavy, 2011; Tarrant, 2018).

Spaces of home and housing have also been of keen interest to feminist geographies, particularly how changing living arrangements under austerity perpetuate and accentuate long-standing structural gendered, racialised and classed inequalities. Research reveals how alterations to housing benefits, in the name of austerity, have forced the sharing of houses by strangers and reframed notions of ‘home’ within welfare policies (Wilkinson and Ortega-Alcázar, 2017). Another example is the ‘bedroom tax’ (a reduction in housing benefit allowance due to so-called under-occupancy), considered one of the most violent policies to emerge from the UK under austerity, ‘disproportionately affecting single people and those couples who find their relationships outside the reconfigured normative values of austerity Britain’ (Brown, 2015: 975; Hall, 2019a). The home is also thought to transform into a space of austerity activism, a site of transformative politics, citizenship and collective action (Jupp, 2017).

Following this, everyday relationships and practices feature heavily in feminist geographical research on austerity in the UK (Hall, 2019b). Recognising the significance of care in the reconfiguring of socio-economic relations under austerity and economic crisis has prompted necessary questions about who cares, how and where, and for whom (Bowlby, 2012; Jupp et al., 2019; McDowell, 2017). Recent work has also looked at changing social relationships in austerity as a space of resistance, including befriending schemes with asylum seekers as an intimate space for everyday political action (Askins, 2015), and gendered practices of thrift during austerity (Holmes, 2019). Everyday relationships with material objects and digital dexterities in austerity has also attracted the attentions of feminist geographers, providing insights into how inequalities and identities become manifest via material and virtual engagements (Bonner-Thompson and McDowell, 2021; Hall, 2020b).

Similarly, everyday embodied encounters in and with austerity are another area of focus for feminist geographies. The affective implications of austerity, such as paranoia (Hitchen, 2019), anticipation (Horton, 2016) and intense atmospheres (Raynor, 2017), speak to the embodied condition and conditioning of austerity (Hitchen, 2016). Such work also highlights multi-scalar impacts of austerity politics on socio-economic relations. Relatedly, feminist geographers have considered the emotional politics of researching personal and economic conditions of austerity, and the politics of fieldwork relationships in such contexts (Hall, 2017).

With this collective body of work, feminist geographies have reasserted their relevance in matters of economy, politics and society, and pervasive socio-spatial inequalities resulting from austerity. Such analysis has deepened understandings of everyday experiences (daily rhythms, routines, relationships, resistances and responsibilities), structural inequalities (the implications of social differences within institutions, finance, politics, communities, families etc.) and multi-scalar socio-economic relations in a time of austerity (within and between global, urban, embodied etc. contexts). Likewise, critical contributions about life in austerity have arguably elevated feminist geographical contributions within the discipline, and within feminist political economy more broadly, leading to a reassessment of spaces of home and family, state and citizen, and productive and reproductive socio-economic relations. In the spirit of advancing and continuing this important work, in the next sections of this paper I outline where else feminist geographies of austerity might go and the generative potential of developing these lines of inquiry.

## IV Feminist geographies of austerity: Five points of intervention

From here, I offer a set of possibilities for advancing debates around feminist geographies of austerity, drawing on long-standing ideas within feminist, economic and cultural theory. Rather than review existing literature, the intention is to outline the conceptual underpinnings already laid, and the potential posed for future work in the fields of social reproduction, everyday epistemologies, intersectionality, voice and silence, and embodied fieldwork. These are five areas with which to take forward feminist geographies of austerity conceptually, empirically and politically. Together, they also recentre and emphasise everyday experiences, structural inequalities and socio-economic relations, all of which are at stake in a time of austerity.

### 1 Social reproduction

Feminist geographers have long been interested in social reproduction and reproductive labour, understood to consist of three, interrelated components: ‘biological reproduction, the reproduction of the labouring population, and provisioning and caring needs’ (Strauss, 2013: 182; Teeple Hopkins, 2015). The concept of social reproduction derives from Marxist analyses of economic activity as consisting of atomised productive and reproductive spheres. Feminist theorists have made significant contributions here. Firstly, identifying how these spheres are highly integrated and must work in tandem for the functioning of the economy at large. And secondly, troubling the privileging of financially profitable activity within understandings of the economy (Federici, 2012; Massey, 2013; Winders and Smith, 2019). Through this, feminist scholarship has encouraged a critical re-tracing of the implications that a focus on paid labour within economic spheres has for understanding care and reproduction *as labour*, extending framings of the economic to include intimacy, love and collective action.

Austerity and social reproduction are also interrelated concepts and conditions; the most pervasive impacts of austerity are ‘in the sphere of reproduction… via cuts to social security benefits, cuts to expenditure on public services, and changes in direct and indirect taxes’ (Pearson and Elson, 2015: 17). This is one of the reasons why women, racialised and working-class communities are left most vulnerable by austerity measures. In addition, austerity across social reproduction has been felt not only as targeted cutbacks, but also in a continued lack of investment and limited reversal of austerity policies (Hall, 2019a; Pearson, 2019). Feminist geographies of austerity and social reproduction have largely been couched in a language of care and domesticity (Jupp, 2017; Strauss, 2013), with opportunities for connecting these everyday experiences to wider structures, economies and labour relations. By revisiting debates on social reproduction – as simultaneously labour, care and intimacy, and the tensions therein – feminist geographies of austerity can be extended in two interrelated ways.

Firstly, social reproduction is a multi-scalar concern, connecting states, economies, households and bodies as sites of labour and ‘terrains of struggle and cultural contestation’ (Massey, 2004; Oswin and Olund, 2010; Silvey, 2012: 6). The lens of social reproduction – when understood as essential to the functioning of the economy (Pearson and Elson, 2015) – also reveals how these scales are interconnected: ‘activities of reproductive care [...] are integral to nation building, [but] for the most part go unrecognised’ (Dyck, 2005: 240; Mitchell et al., 2003).

There are opportunities for feminist geographies of austerity to take a multi-temporal and multi-spatial perspective using the lens of social reproduction, to identify how the histories, forms and possibilities of reproductive labour have been shaped by and with austerity. This involves situating the impacts of austerity not only contemporaneously but tracing this to other global-intimate socio-economic practices and flows. This might include charting links with colonial, international and migratory pasts and presents that give rise to nuanced understandings of racialised, gendered and classed labour within both waged and unwaged economies of care (see examples in the US context on health workers by England and Henry, 2013, and domestic workers by Mullings, 2009). It could also include drawing connections to socio-economic trends around neoliberalism, state intervention, welfare retrenchment and austerity, and the place of social reproduction within long-term policy-making.

Working through these space-times of social reproduction in the UK context can help reveal how individualised experiences are entwined with global mobilities, intersectionalities, identities and relationalities (Mullings, 2009), reconnecting definitions of care as both embodied and generational labour. Understanding the legacies of austerity and social reproduction, as told through histories and everyday encounters with migration, labour, welfare, nationalism and so forth, also encourages us to revisit meanings of labour as emplaced across space-time (Henry, 2015; McDowell, 2015; Mitchell et al., 2003). Gendered and racialised reproductive labour within care institutions, such as the UK National Health Service (NHS) is one example. The NHS has faced substantial cuts under austerity (Pearson and Elson, 2015). The NHS employs more women than men, and women also make up over half of hospital admissions (Hall et al., 2017; WBG, 2020). Over a fifth of the NHS workforce are non-white (GOV.UK, 2021), and Black, Asian and Minority Ethnic groups as recipients are reported to be most likely to live with chronic and long-term illness (Hall et al., 2017). Thinking through these emplaced labour space-times, then, the heightened impact of healthcare cuts on people of colour (as both providers and recipients) sits in parallel with an abiding rhetoric of ‘abuse’ of public services by immigrants (see Erel, 2018; Nowicka, 2020). These issues are threaded into later sections on intersectionality, and voice and silence.

There are links here to non-domestic practices and relations, as the second strand I want to highlight. A focus on social reproduction in austerity emphasises the sheer interconnectedness of productive and reproductive economies, particularly though the example of care work beyond home or family. Social reproduction has been described as a patchwork of activities, a mixed economy of formal and informal sites of labour, interconnected within the economy and individual practices (Power and Hall, 2018). Feminist geographies have provided insights into the spatial variance of social reproduction, as well as relational dynamics of care work as paid and public labour, with global examples including the labour of surrogates (Lewis, 2018), live-in care workers (Schwiter et al., 2018), gang labourers (Strauss, 2013) and nurses (Henry, 2015).

Through disentangling these formal/informal, domestic/non-domestic, paid/unpaid reproductive relations in austerity, power and inequality may be further revealed. Coming back to the examples of health and social care, austerity cutbacks have led to the revision of spaces and responsibilities of care, and so too informality and profit. With the retrenchment of public spending, responsibilities for social and childcare have retreated into domestic spaces (Jupp, 2017). Simultaneously, paid and formalised care labour has become further stratified according to gender, class and race, and reliant on cheaper labour. Authors writing from the US context describe this as an example of stratified commodified care (Henry, 2015; Teeple Hopkins, 2015), which fuzzies the often too-boldly drawn boundaries between reproductive and productive labour. Feminist articulations of the uneven distribution of reproductive labour can thus help to shed light on the complexities of care, intimacy and emotional work in the context of austerity cuts. This is an analytical lens that should be extended to feminist geographies of austerity in the UK, with a particular need to ‘methodologically centre race in analyses of unpaid [and paid] labour’ (Teeple Hopkins, 2015: 136). This involves revisiting assumptions around care as intimate and re-complicating the relationship between emotional and embodied labour (Twigg, 2006).

Taking feminist geographies of austerity forward with this dual focus on the space-times of social reproduction, and re-complicating the relationship between productive and reproductive spheres of care, will no doubt also elicit detailed discussions about the place of social reproduction, in daily life and in the discipline. Feminist geographies are key in articulations of power relations across space and scale, including how these relations shape and are shaped by epistemic politics, knowledge and norms, to provide critical accounts of social reproduction in, and in spite of, austerity. This also serves to synthesise and propel intellectual contributions on everyday experiences, structural inequalities and multi-scalar socio-economic (and temporal) relations, including at their intersections. These themes are continued throughout the remaining sections, where the discussion now turns to politics of everyday epistemologies.

### 2 Everyday epistemologies

Feminist epistemologies are overwhelmingly concerned with everyday experiences, spaces and politics, whilst challenging structural and macro-cultural processes of knowledge formation. In practice, feminist epistemologies attempt to move away from gender-coded practices, and ‘rather than taking for granted, the bodily dimensions of women’s activities’ (McDowell, 1992: 411), attempt to understand the structures and spaces (such as productive and reproductive economies) in which knowledge and bodies are co-constituted (Rose, 1993). The notion of an emplaced, embodied feminist epistemology, grounded in experience and subjectivity, has influenced within geography a whole raft of embodied and emplaced methods (Longhurst and Johnston, 2014), reflexive discussions of positionality (Rose, 1997), and ‘ways of knowing’ (Nast, 1994: 55). These ideas are often attributed to Haraway’s (1988: 581) concept of ‘situated knowledges’:

‘[a] politics and epistemologies of location, positioning and situating, where partiality and not universality is the condition of being heard [...] a view from a body, always a complex, contradictory, structuring and structured body, versus the view from above, from nowhere, from simplicity’ (ibid, p. 589).

With this section I introduce and challenge dominant epistemological positionings with policy and popular UK discourse in the context of austerity. Through examples of everyday ‘coping’, ‘strategies’ and ‘resilience’, I reveal how everyday epistemologies concerning austerity coalesce around individualised political actions, agency and projections. Here, individuals are presumed to hold capacity to respond to structural inequalities of poverty, insecurity and disadvantage. In response, I suggest how feminist geographers can assemble and articulate more grounded, collective and embodied epistemological framings of austerity.

Everyday life in austerity is often described a form of hardship; characterised by struggle, inequality and insecurity. Across the growing body of social policy and research exploring lived experiences of austerity, multiple references are made to ‘coping’, 'strategies' and ‘resilience’ (Harrison, 2013). These are commonplace signifiers that do more than simply describe: they come to define, characterise and homogenise lived experiences of austerity and, significantly, reactions and responses to it. They seep into academic language, characterising people as developing some sort of ‘strategy’ to deal with everyday, acute hardships (Bondi and Christie, 2000; Cappellini et al., 2014; Frade and Coelho, 2015; Thomson et al., 2010). Whilst likely well meaning, such phraseology is innocuous and rarely unpacked. Furthermore, these epistemic interpretations of austerity tend to be imposed from ‘above’, thus, sitting at odds with feminist epistemologies that start with the body and relational space.

The use of ‘coping strategies', for instance has long been argued to raise questions about the privilege and position of research, with the contention that the term is ‘patronising’ (Crow, 1989: 20), masculinist and ‘imputes objectives which the individuals within a situation themselves would not recognise’ (Edwards and Ribbens, 1991: 480; also see Morgan, 1989). There are similarities with debates on resilience and austerity, whereby ‘there is a tendency towards value judgements on what is “good coping”’ (Harrison, 2013: 99); shifting responsibility for economic insecurity from government to individuals and marginalised groups.

Emerging writings within feminist geographies of austerity have provided much-needed insights into the gendered experiences of living in and through austerity (see Section 3), showcasing an everyday epistemological approach of theorising from the body and personal experiences (Hall, 2017; Hitchen, 2019; Stenning, 2018; Wilkinson and Ortega‐Alcázar, 2019). Deploying written or spoken language to depict personal experiences can be tricky, with distinct limits to conveying embodied, situated knowledge (Davidson et al., 2012). Critiques of forms of imposed knowledges (coping, strategies, resilience), as a means of representing everyday experiences of austerity, show that for people and communities struggling, trying (and sometimes failing) to ‘cope’ is a perpetual state of precarity and uncertainty (Raynor, 2017; Thomson et al., 2010).

Alternative phrases used in the UK context include ‘getting by’ (Harrison, 2013; Holmes, 2019), ‘getting on’ (Hitchen, 2016; Holmes, 2019), ‘ploughing on’ (Wilkinson and Ortega-Alcázar, 2019) and ‘ways of coping’ (Edwards and Ribbens, 1991). Together, these alternatives arguably promote an epistemological framework that centres the experiences of those most affected. And while these might not be as catchy as ‘coping strategies', they make space for a grounded approach that more closely reflects how those living in and through austerity might comprehend their own experiences.

Deconstructing the epistemological framing for everyday experiences of austerity is a political task that works to directly expose and question underlying epistemological assumptions. This is about more than representation; it concerns the epistemological knowledges assumed and imposed on individuals about lived experiences of austerity, and how those knowledges perpetuate ideas about the extent to which individuals are responsible for their own reckoning. From a feminist epistemological perspective, to talk of coping, strategising and mitigating is universalising and impersonal, situating often the most vulnerable as both the problem and the solution. To push back and question these epistemological framings is to also resist austerity as a personal condition, revealing entanglements of practices, relations and interdependencies, not least with the state (Bruff and Wöhl, 2016; Harrison, 2013; Thomson et al., 2010). This confirms long-held feminist geographical arguments that the everyday is a personal-political space of resistance, activism and collectivism (Dyck, 2005; Hall, 2020a; Hanisch, 1970).

This is a political challenge for feminist geographers to grasp policy-public epistemologies of austerity more firmly; to acknowledge them as epistemic challenges; and to develop stronger responses. This may involve tracing the usage and propagation of such terminology as coping, strategy, resistance, exposing these as self-serving epistemological stances. Revisiting personal or public data archives with this in mind could reveal alternative ways in which people talk about and frame everyday epistemologies of austerity. From this, I suspect, there would be an array of examples from which to rework epistemological positions to be more attuned with everyday experience of austerity; which convey plurality, complexity and heterogeneity; and that emanate from the body (see Stenning, 2018; Wilkinson and Ortega-Alcázar, 2019). This work could help to articulate how lived experiences of austerity are wrapped up in a host of embodiments, subjectivities and positionalities.

Another possibility is to place energies into research projects that actively translate for academic, policy and public audiences the concerns and experiences of participants as a legitimate epistemological position; that of lived experiences of austerity *as* feminist epistemology (see 4.4 and 4.5). The next section extends these points conceptually, to explore how socio-economic responsibilities and labours in austerity might be understood through a feminist intersectional framework.

### 3 Intersectionality

Geographical writings on intersectionality highlight its potential to reconnect everyday experiences with structural inequalities that undergird multi-scalar socio-economic implications of austerity. From the foundational work of Black and Critical race scholars, intersectionality is a framework for systematically exposing the ‘multiple axes of differentiation’ when comprehending the structural inequalities that mete out in everyday life (Brah and Pheonix, 2004: 76). Crenshaw (2018) has recently referred to intersectionality as a framing device, simultaneously theory, analytic and method (Lewis, 2013; McCall, 2005; Windsong, 2018). This approach, working from points of difference, is also often associated with third-wave feminism and a move away from homogenising women’s lived experiences and identities, despite sometimes being a means of effective organising (Federici, 2012).

Feminist geographers with expanding interests in intersectionality have explored entanglements of gender, race and ethnicity, class, disability, alongside other social categories. Described as a tool to expose how ‘space and identities are co-implicated’ (p. 19), intersectional frameworks trouble how ‘some categories such as gender might unsettle, undo, or cancel out other categories such as sexualities’ (Brown, 2012; Valentine, 2007: 15; Valentine 2008). Recent agendas in geographical research include ‘to advance how intersectionality is theorized, applied in research and used in practice’ (Hopkins 2019: 942), and ‘to work collaboratively with practitioners to do so’ (Hopkins, 2019: 944; also see Bauer, 2014; Hopkins and Pain, 2007). Advancements also include intersectionality as method, given critiques of a lack of methodological direction (Baylina Ferré and Rodó De Zárate, 2016; see Coddington, 2017; Hopkins, 2018; Johnson, 2020; Raghuram, 2019; Valentine, 2007; Yuval-Davies, 2006).

Concerns have, nonetheless, been raised as to the application of intersectionality within geography, particularly in terms of representation. This includes greater acknowledgement of ‘the activist origins of intersectionality’ (Hopkins, 2018: 586); the fetishising or ‘white washing’ of intersectionality by White European feminism and feminist agendas (Rodó-de-Zárate and Baylina, 2018: 550); cautions against an adding-and-stirring approach (Bonds, 2013; Pollard, 2013); and how the removal of race from intersectional accounts is a form of epistemic violence (Mollett and Faria, 2018). There are also many instances of feminist geographical work that explores the intersectional nature of social categories and difference (Binnie, 2011; Kobayashi and Peak, 1994; McDowell, 1991), with claims that these ‘intersectionality-like’ contributions can be overlooked (Hopkins, 2018: 586). Also, feminist geographical engagements with intersectionality are themselves spatially varied, with scholars in the US in particular engaging with how intersectional and Black experiences structure socio-economic relations (Summers, 2019; Werner et al., 2017).

As for intersectionality and austerity in the UK, a growing body of interdisciplinary feminist contributions are notable. Scholars have explored gender, race and class in the case of ‘the effects of austerity on minority women’s vulnerability as well as their activism and new solidarities created by and for them’ (Bassel and Emejulu, 2014: 131). This includes work on cuts to reproductive spheres of healthcare, welfare, social care and public service investments (see 4.1; Bassel and Emejulu, 2017; Erel, 2018; Pearson and Elson, 2015). There are also critiques of austerity that consider the intersectional nature of social categories, but do not refer to intersectionality as such. This spans a wide range of themes, from the sexual politics of the bedroom tax (Brown, 2015); to classed, gendered and generational experiences of austerity (McDowell, 2012); cross-cutting gendered and health inequalities (Greer-Murphy, 2017); and how welfare cuts have affected lesbian, gay and bisexual people (Beckett, 2015).

However, I hesitate in labelling these and similar works as adopting an ‘intersectionality-like’ analysis (Hopkins, 2018: 586). While there have been calls for geographers to expand intersectional analysis beyond the ‘trinity’ of gender, race and class (Brown, 2012 545; Di Feliciantonio and Brown, 2015; Valentine, 2007), in the instance of intersectionality and UK-based austerity, race is more likely to be masked than gender or class within such approaches (Phinney, 2020; Yuval-Davies, 2006). Such omissions thus have the potential to lead to analyses of austerity that could better be described as ‘intersectionality-light'.

There is, therefore, much scope for greater engagement by feminist geographers on the relationship between austerity and intersectionality and these ‘axes of difference’ (Dyck, 2005: 242). Such insights – both at an everyday, personal scale and across local, regional and (inter)national scales – could be used to question and actively address the injustice that certain people and groups are more acutely impacted by austerity. Further comparative work, whether cross-spatial (e.g. national or international comparisons) or cross-temporal (e.g. archival work, oral histories, intergenerational exchanges), would also reveal the interactions between intersectionality and space-time. Indeed, a geographical approach to intersectionality is one that simultaneously pays attention to the social and the spatial (Greer Murphy, 2017; Mollett and Faria, 2018; Valentine, 2007), that is at once ‘intersectional, grounded, [and] place-based’ (Di Felicinantonio and Brown, 2015: 966). To appreciate uneven experiences of austerity, from everyday economic practices to regional variations, feminist geographical analysis needs to consider how austerity affects different people, groups and communities, in different ways, according to social-spatial locations and positions.

There are also opportunities to extend intersectionality as method within feminist geographies of austerity. This, in line with what others suggest, involves more than simple, oft-unchallenged ‘declarative statements’ as to one’s own positionality (Johnson, 2020: 91), or using an ‘additive approach’ (Hopkins, 2019: 938) but to actively examine structures via method. Participatory methods, co-production, visual methods, autoethnography, case studies and dialogue have all been mooted. With such knowledge generation, shared narratives of everyday experiences and emplaced power relations, intersectional analyses might be achieved (Baylina Ferré and Rodó De Zárate, 2016; Hopkins, 2018; McCall, 2005; Raghuram, 2019; Sircar, 2021).

Overarchingly, an intersectional methodological approach involves challenging the institutional structures of whiteness, middle-class and masculine identities by/in/across these processes (Johnson, 2020), and to see this as the start of a conversation, rather than an easy ‘fix’. This can, I suggest, be applied across the breadth of research design, methodology and empirical approaches to austerity; to include research staff hirings, choice of fieldwork locations, sampling and recruitment, reflexive subjectivities, ethical considerations, choice of methods, language translation, points of analysis, forms of data sharing and so forth. The next two sections contribute to fleshing out some of these critical approaches, by looking at voice and silence, and embodied fieldwork.

### 4 Voice and silence

As highlighted in sections 2 and 4.2, feminist scholars have long made the case for ‘giving voice’ as a route to recognition and empowerment, and the role of collaboration, activism and solidarity in this endeavour. Representing, platforming and amplifying the experiences of marginalised groups (Coddington, 2017; Parizeau et al., 2016), as well as creating safe spaces for listening (Hanisch, 1970; Hyams, 2004), are avenues to achieve this. But there are also words of warning about what ‘giving’ voice entails; who has the power, position and privilege to do this ‘giving’, and who ultimately is ‘listening’ – and with what effects? Wrestling with the contradictions that feminist approaches might behold, it is vital not to *speak for* others, to represent without appropriating, and for allyship to be a force for meaningful action (England, 1994; also see Gayle, 2020; McDowell, 1991; Oakley, 1981; Ortega, 2006). This section moves away from ‘giving’ voice towards deeper consideration of voicing and silence within feminist geographies of austerity.

There are two significant ways in which feminist scholars have mobilised the voices of women and marginalised groups affected by austerity; in the presentation of data, and through public engagement activisms. On the first, data featured within academic and policy literature showcases the strengths of amplifying lived narratives to influence agendas, debates, policy and practice; asking, observing and representing these experiences *in their own words*. As such, the issue of voice, when approached via a feminist lens, also draws together the overarching themes of this paper; everyday experiences, structural inequalities and multi-scalar socio-economic relations.

Rich empirical engagements with austerity in the UK include the experiences of migrant women and community activists (Jupp, 2017), ethnographic research with staff and customers at an affected library service (Hitchin, 2019), and interviews with practitioners, young people, parents and carers at community centres (Horton, 2016). Garnering highly personal responses often necessitates in-depth, qualitative empirical work, making space and time for experiences to be shared and fully represented. Nonetheless, as Hyams (2004: 109) points out, silences are equally ‘meaning-ful’ in how they contribute ‘to the flow and direction of the interaction and meaning-making process'.

Creative ways for amplifying lived experiences of austerity have also started to emerge as a means for feminist scholars to share findings about the many everyday injustices of austerity, arguably themselves a form of quiet, affective activism (Gayle, 2020; Jupp, 2017). These outputs, often targeted at non-academic audiences, go beyond documentation to act as political tools to campaign, influence public debates and speak ‘up’ to policy (see section 2). Examples include zines (Hall and Stringer, 2017; Welfare Imaginaries Zine, 2020); co-produced animations (Hall et al., 2017; Jupp, 2021); graphic stories (Taylor, 2020); and collaborative theatre productions (Gray and Smith, 2017; Raynor, 2017). In these examples, creative outputs engage wider publics to challenge dominant and stigmatising discourses of blame and exclusion.

While noteworthy for the connections these outlets offer between feminist academic debates, public discourses and political action regarding austerity in the UK, the focus is invariably on that which can be heard and thus documented. Moving away from well-worn discussions about ‘giving voice’ and instead towards greater understanding of quietness, silence and silencing can open up avenues for rethinking voice *in* and voic*ing* austerity. This is partly about enacting the everyday epistemologies outlined in 4.2, and developing intersectional methodologies, as per 4.3. A growing body of literature on quiet politics, spearheaded by feminist geographers, reveals how in searching for the fireworks, the louder political proclamations, everyday political acts often go unnoticed (Askins, 2015; Horton and Kraftl, 2009; Jupp, 2017; Pottinger, 2017). Most of these authors also write from the context of austerity, where participants have been at the sharp end of public spending cuts and find themselves seeking refrain, companionship and hope (Wilkinson and Ortega-Alcázar, 2019).

It is here that feminist engagements with silence and silencing in austerity can contribute. Everyday micro-aggressions – exclusions, hostility and confrontations – are forms of quiet politics that can be subtle in their presence and performance (Coddington, 2017; Parizeau et al., 2016). Micro-aggressions have the potential to become more pronounced in austerity contexts, in spaces where power differentials according to gender, race and class, citizen and state, giver and receiver, are enhanced and can be fraught, for example job centres, food banks, libraries, shopping centres, language classes and post office queues (Hall, 2019a; Robinson, 2020). These are all spaces for further investigation.

Moreover, micro-aggressions can also be understood as a politics of silenc*ing*. As Domosh (2015) explains ‘the words that recognise and speak back to these micro-aggressions are difficult to conjure; a rebuke does little good since the insult wasn’t “intended”, while a complaint raises the specter of the “sensitive and difficult person”’. Joshi et al. (2015: 317), writing on whiteness and invisible aggressions, note that micro-aggressions ‘are difficult to pinpoint’ by virtue of their sheer passivity, but can lead to the internalisation of visceral reactions. It is also important to consider how ‘bodies are the surfaces upon which micro-aggressions and differences are played out’ (Hall, 2019a: 157; Torres, 2018), whether as ‘a facial expression of disgust, or a bodily expression of distancing or withdrawal’ (Krumer-Nevo, 2017: 6; Gayle, 2020; Skop, 2015). And while intimate and micro-scale (Torres, 2018), micro-aggressions can nevertheless be understood as ‘the embodiment of broader narratives and structural inequalities that occur at many other scales’ (Joshi et al., 2015; Mullings et al., 2016; Skop, 2015: 433).

There is therefore much scope for feminist geographies to take further scholarship on the spaces, relations, scales and substances of voices, silences and silencing in austerity. This work would make important contributions to understanding austerity as everyday experiences (silences in daily life, intersections and practices), structural inequalities (experiences missing or mis-represented, and why), and multi-scalar socio-economic relations (from personal acts to state policies). This might include where and by whom silence and silencing are enacted and felt, and what austere conditions mean for how voicing and silencing practices are addressed. It could also include turning attentions to methodologies for listening to, or sitting with, silence; slower, gentler paced observational methods that focus on ‘being with’ (Askins, 2015; Pottinger, 2020); techniques such as oral history and biographical methods that make space for silences and omissions as meaningful (Geiger, 1990; Sangster, 1994); and activist-ethnographic methods that explicitly develop engagements with everyday and structural political struggles (Leyva del Rio, 2021); whilst being attuned to critiques of voyeurism when researching lived experiences (England, 1994; Stacey, 1988).

These considerations also extend to silence and silencing within academic debate, through micro-aggressive practices of citational exclusions and peer reviewing, and data gaps, re-appropriation and misrepresentation (Ahmed, 2017; Davies and O'Callaghan, 2014; Gayle, 2020; Ortega, 2006; Werner et al., 2017). The next section builds on these discussions by working through politics and ethics of embodied fieldwork.

### 5 Embodied fieldwork

In ‘Bodies, gender, place and culture: 21 years on’, Longhurst and Johnston (2014) identify ‘embodied fieldwork and methodologies’ as a space ‘for growth’ within the discipline (p. 271–272). Geographers have described the body as a sort of fieldwork equipment, a tool for data collection, or ‘instrument of research’ (Crang, 2003; Longhurst et al., 2008). Feminist researchers have noted feelings of discomfort and fear around participants (Silvey, 2003), developing friendships (Blake, 2007), awkward encounters when disengaging or leaving the field (Hall, 2014), grief (Falconer, 2017; Stacey, 1998) and so on. During fieldwork, as in all everyday encounters, bodies of researchers and participants are ‘placed’ according to gender, class, race, age, sexuality, disability, etc. (Skeggs, 2004), including where they intersect (see 4.3). Embodied fieldwork in austerity is the final theme I highlight as having potential for further examination, drawing together earlier discussions on care and reproduction, epistemology, intersectionality, and voice and silence.

Feminist geographies of austerity have touched on corporeal connections within fieldwork, providing detail on everyday, intimate relations in austerity. For instance ‘the day-to-day existence of coping with welfare reform’ has been described as often leading to ‘fatigue, a gradual slow wearing out that comes with having to endure everyday hardship’, revealing how ‘weariness is an integral part of understanding austerity’s everyday affects on the body’ (Wilkinson and Ortega-Alcázar, 2019: 157). Working with entanglements of psychic and social dynamics, the sensation of being ‘squeezed’ (Stenning, 2018) has been deployed to illustrate how austerity is simultaneously emotional and embodied, and experiences such as food poverty as literal ‘hunger pains’ (Strong, 2021). And the process of researching austerity has been conceptualised as a type of physical labour, bodywork and form of care work (Hall, 2017).

Seeking to problematise the binary of ‘familiarity’ and ‘difference’, feminist geographers have also shown that while at times problematic, embodied similarities and positionalities can be a site for building rapport (Falconer, 2017; Richardson, 2015; Tarrant, 2014), granting access to participants and groups in ways that another body, any*body* might not. Wilkinson (2016: 117) argues for an extended discussion ‘of the appearance of the researcher beyond meta-categories such as gender and skin colour’, to include similarities in ‘“embellishments”; for instance: make-up, hair extensions, fake nails and false eyelashes'. Flipping Wilkinson’s (2016) proposition, fieldwork relationships can also be built on bodily *difference*. Examples include the navigation of intersecting generational and gender differences in research on grandfathering (Tarrant, 2014), and differences of sexuality and faith in research with conservative families (Sou, 2021). Fieldwork can thus serve as a reminder of how the body provides a means of socio-spatial locatedness (Longhurst, 1994).

Given the protracted nature of austerity in the UK – 10 years and counting – bodily changes and transformations during fieldwork can be considered in a new light, including changes with time *on* the body. This might look at how institutional environments can bear embodied impacts for researchers, and the limits placed on temporalities, spatialities and relationalities of embodied fieldwork. For instance austere funding environments, acutely impacted (even saturated) social groups as research participants, and the seeping of austerity into everyday academic personal and professional life (Christopherson et al., 2014; Davies and O’Callaghan, 2014; Hall, 2017) may lead to new challenges and opportunities for embodied methodologies. Contemporary forms of academic precarity (predicated on hyper mobility, productivity, rapid outputs, etc.) also form part of these discussions, and the challenges such conditions may present for developing and maintaining embedded, meaningful research relationships. These also become challenges for tracing the long-term impacts of austerity on specific groups via embodied approaches, including the longitudinal inflictions of neoliberal processes of welfare dismantling and experiences during and after the current Coronavirus pandemic.

With obstacles to documenting change in and with austerity via embodied fieldwork approaches also comes scope for feminist developments in different ways of doing embodied fieldwork in austerity. One possible response to strained socio-economic conditions presented by austerity is ‘patchwork ethnography’. Connecting with discussions in 4.2 on positioning and 4.4 on pace, patchwork ethnography is ‘designed around short-term field visits, using fragmentary yet rigorous data’ to ‘maintain the long-term commitments, language proficiency, contextual knowledge, and slow thinking that characterizes so-called traditional fieldwork’ (Günel et al., 2020). Bearing similarities with episodic ethnography (Hall, 2014), patchwork ethnography also responds to concerns about ‘neoliberal university labour conditions’ (Günel et al., 2020). As such, it speaks to the three overarching themes developed throughout this paper, attending to daily experiences of fieldwork, structural inequalities within personal and professional realms, and uneven relations across intimate and institutional spaces in austerity.

Added to this, embodied fieldwork can refer as much to bodily experiences of, in and with fieldwork (encompassing other people, materials, living things, emotions, etc.), as to embodied experiences before and after fieldwork encounters. Referred to as ‘the temporal phases of qualitative research’, emotionally inducing, closely embodied fieldwork can have its own ‘afterlife’, for which subtle, flexible and compassionate ethical approaches are needed (Pascoe Leahy, 2021: 1). Furthermore, the makings and endings of austerity provide a unique set of conditions for feminist researchers, raising questions about the pace, tone and content of fieldwork as it sits in/out of sync with the everyday lives of those affected (Hall, 2019a), as well as absences and losses (Raynor, 2018). I come back to these legacies of austerity in more detail below.

## V Conclusions

An overview of the contributions of feminist scholars and activists reveals a palpable vibrancy of critique and resistance regarding the divisive and gendered nature of austerity cuts in the UK. This paper takes this agenda further, offering five interconnected points of intervention aimed at expanding the conceptual, empirical and political framing of feminist geographies of austerity. Firstly, I argue for revisiting social reproduction as a lens to view multi-scalar and beyond-domestic geographies, to develop deeper understandings of space-times of reproduction in austerity. Secondly, I show how feminist epistemologies of austerity might be more closely considered, to reshape and resist dominant framings that place responsibility to ‘cope’ onto individuals. Thirdly, I identify scope for feminist geographies to engage more meaningfully with intersectionality as a framework to unveil the socio-spatial impacts of austerity. The fourth intervention focused on the possibilities of silencing in and of austerity: the politics of difference enforced through austerity (quietly and subtly), and the effect austerity policies can have on recipients and debates (quieting and silencing). Lastly, embodied fieldwork was highlighted, in terms of how the protracted and pervasive nature of austerity results in changes on bodies during fieldwork and academia more broadly.

Throughout these interventions I have pronounced their generative potential in advancing debates and developing avenues for feminist geographies of austerity in the UK and beyond. Together, they coalesce around three overarching themes: everyday experiences, structural inequalities and socio-economic relations. Within these themes – focused on the interlocking social, spatial, temporal and relational impacts of austerity – feminist geographies have underscored the inequalities, differences and politics raised by austerity, as connected to wider processes of state retrenchment, welfare reform and neoliberalism. As a result, these themes help reconfigure geographical attentions towards the deep and acute implications of austerity, and to what is at stake: inequality, care, social relationships, social reproduction, welfare, and poverty and disadvantage.

Together, these are significant areas for feminist scholarship, not least because austerity remains a contentious and pertinent issue in the UK, as well as across Europe, North America, Latin America, Southern Africa and Australasia. This enduring relevance has been compounded by the ongoing COVID-19 pandemic. The slowing and effective stopping of many parts of the economy have created the conditions for a predicted economic recession in the UK and ongoing austerity measures (Gopinath, 2020). Simultaneously, the ability of health and social care services to adequately respond to the pandemic has been hindered by a decade of austerity cuts (Flesher Fominaya, 2020), leaving significant numbers of people (with racially minoritised groups disproportionally represented) living in poverty and more vulnerable to contracting the virus due to ill health, substandard housing and low paid employment opportunities (Craddock, 2020; Gillespie and Hardy, 2020). Emerging reports also point to the gendered nature of the everyday impacts of the pandemic, including concerns around inequalities in care responsibilities, higher likelihood of exposure to COVID-19, and increases in reports of domestic violence (WBG, 2020). And austerity of course already has a legacy, in the life-courses and generations of those at the sharpest end of spending cuts over the last decade (Hall, 2021). It is imperative then, that feminist geographies play a key role in offering critiques of, insights about and resistance to austerity, starting with the interventions outlined here, because austerity will not be going away any time soon.

## References

[bibr1-03091325211065118] Ahmed S (2017) Living a Feminist Life. Croydon: Duke University Press.

[bibr2-03091325211065118] Askins K (2015) Being together: everyday geographies and the quiet politics of belonging. ACME: An International E-Journal for Critical Geographies 14(2): 470–478.

[bibr3-03091325211065118] Bassel L Emejulu A (2014) Solidarity under austerity: intersectionality in France and the United Kingdom. Politics & Gender 10(1): 130–136.

[bibr4-03091325211065118] Bassel L Emejulu A (2017) Minority Women and Austerity: Survival and Resistance in France and Britain. Bristol: Policy Press.

[bibr5-03091325211065118] Bauer GR (2014) Incorporating intersectionality theory into population health research methodology: challenges and the potential to advance health equity. Social Science & Medicine 110: 10–17. 24704889 10.1016/j.socscimed.2014.03.022

[bibr6-03091325211065118] Baylina Ferré M Rodó de Zárate M (2016) New visual methods for teaching intersectionality from a spatial perspective in a geography and gender course. Journal of Geography in Higher Education 40(4): 608–620.

[bibr7-03091325211065118] Beckett C (2015) Assessing the cost of cuts in welfare spending for lesbian, gay and bisexual people. Social Policy and Society 14(1): 29–41.

[bibr8-03091325211065118] Binnie J (2011) Class, sexuality and space: a comment. Sexualities 14(1): 21–26.

[bibr9-03091325211065118] Blake MK (2007) Formality and friendship: research ethics review and participatory action research. Acme 6(3): 411–421.

[bibr10-03091325211065118] Bondi L Christie H (2000) Working Out the Urban: Gender Relations and the City. Oxford: A Companion to the CityBlackwell, pp. 292–306.

[bibr11-03091325211065118] Bonds A (2013) Racing economic geography: the place of race in economic geography. Geography Compass 7: 398–411.

[bibr12-03091325211065118] Bonner-Thompson C McDowell L (2021) Digital geographies of austerity: young men’s material, affective and everyday relationships with the digital. Geoforum 120: 113–121.

[bibr13-03091325211065118] Bowlby S (2012) Recognising the time—space dimensions of care: caringscapes and carescapes. Environment and Planning A 44(9): 2101–2118.

[bibr14-03091325211065118] Bradley H (1986) Work, home and the restructuring of jobs. In: Purcell K Wood S Waton A , et al. (eds), The Changing Experience of Employment: Restructuring and Recession. London: Macmillan, pp. 95–113.

[bibr15-03091325211065118] Brah A Phoenix A (2004) Ain’t I a woman? Revisiting intersectionality. Journal of International Women’s Studies 5(3): 75–86.

[bibr16-03091325211065118] Brah A Szeman I Gedalof I (2015) Introduction: feminism and the politics of austerity. Feminist Review 109: 1–7.

[bibr17-03091325211065118] Brown M (2012) Gender and sexuality I: intersectional anxieties. Progress in Human Geography 36(4): 541–550.

[bibr18-03091325211065118] Brown G (2015) Marriage and the spare bedroom: exploring the sexual politics of austerity. ACME: An International Journal for Critical Geographies 14(4): 975–988.

[bibr19-03091325211065118] Bruff I Wöhl S (2016) Constitutionalizing austerity, disciplining the household. In: Hozic AA True J (eds), Scandalous economics: Gender and the politics of financial crises. Oxford: Oxford University Press, pp. 92–108.

[bibr20-03091325211065118] Cappellini B Marilli A Parsons E (2014) The hidden work of coping: gender and the micro-politics of household consumption in times of austerity. Journal of Marketing Management 30(15–16): 1597–1624.

[bibr21-03091325211065118] Chant S (1997) Women-headed Households: Diversity and Dynamics in the Developing World. Basingstoke: Palgrave Macmillan.

[bibr22-03091325211065118] Christopherson S Gertler M. Gray M (2014) Universities in crisis. Cambridge Journal of Regions, Economy and Society 7: 209–215.

[bibr23-03091325211065118] Coddington (2017) Voice under scrutiny: feminist methods, anticolonial responses, and new methodological tools. The Professional Geographer 69(2): 314–320.

[bibr24-03091325211065118] Craddock E (2017) Caring about and for the cuts: a case study of the gendered dimension of austerity and anti‐austerity activism. Gender, Work & Organization 24(1): 69–82.

[bibr25-03091325211065118] Craddock E (2020) ‘”We’re All in it Together?” Austerity, COVID-19 and Persistent Inequalities’. Discover Society. https://discoversociety.org/2020/04/09/were-all-in-it-together-austerity-covid-19-and-persistent-inequalities/.

[bibr26-03091325211065118] Crang M (2003) Qualitative methods: touchy, feely, look-see? Progress in human geography 27(4): 494–504.

[bibr27-03091325211065118] Crenshaw K (2018) From shattered ceilings to a broken democracy: the post-racial condition of trump’s America. In: British Sociological Association Annual Conference 2018 - Plenary, Newcastle-upon-Tyne, UK, 10th April 2018. Northumbria University.

[bibr28-03091325211065118] Crow G (1989) The use of the concept of “strategy” in recent sociological literature. Sociology 23(1): 1–24.

[bibr29-03091325211065118] Davidson J Smith MM Bondi L (eds), (2012) Emotional Geographies. Ashgate Publishing, Ltd.

[bibr30-03091325211065118] Davies H O'Callaghan C (2014) All in this together? Feminisms, academia, austerity. Journal of Gender Studies 23(3): 227–232.

[bibr31-03091325211065118] Di Feliciantonio C Brown G (2015) Introduction: the sexual politics of austerity. ACME: An International Journal for Critical Geographies 14(4): 965–974.

[bibr32-03091325211065118] Domosh M (2015) How we Hurt Each Other Every Day and What we Might Do about it. Association of American Geographers Newsletter. http://news.aag.org/2015/05/how-we-hurt-each-other-every-day/(accessed 14 December 2018).

[bibr33-03091325211065118] Dyck I (2005) Feminist geography, the ‘everyday’, and local-global relations: hidden spaces of place-making. The Canadian Geographer 49(3): 233–243.

[bibr34-03091325211065118] Edwards R Ribbens J (1991) Meanderings around `Strategy': a research note on strategic discourse in the lives of women. Sociology 25(3): 477–489.

[bibr35-03091325211065118] Elson D (1998) The economic, the political and the domestic: businesses, states and households in the organisation of production. New Political Economy 3(2): 189–208.

[bibr36-03091325211065118] England KVL (1994) Getting personal: reflexivity, positionality, and feminist research∗. The Professional Geographer 46(1): 80–89.

[bibr37-03091325211065118] England K Henry C (2013) Care work, migration and citizenship: international nurses in the UK. Social & Cultural Geography 14(5): 558–574.

[bibr38-03091325211065118] Erel U (2018) Saving and reproducing the nation: struggles around right-wing politics of social reproduction, gender and race in austerity Europe. Women’s Studies International Forum 68: 173–182.

[bibr39-03091325211065118] Falconer E (2017) Moments of collusion? Close readings of affective, hidden moments within feminist research. Women's Studies International Forum 61: 75–80.

[bibr40-03091325211065118] Fawcett Commission (2012) The Impact of Austerity on Women. London: Fawcett Society Policy.

[bibr167-03091325211065118] Federici S (2012) *Revolution at Point Zero: Housework*, *Reproduction, and Feminist Struggle**. *PM Press*.*

[bibr41-03091325211065118] Ferber MA Nelson JA (eds), (1993) Beyond Economic Man: Feminist Theory and Economics. Chicago: University of Chicago Press.

[bibr42-03091325211065118] Flesher Fominaya C (2020) How Austerity Measures Hurt the Covid-19 Response. Oxford University Blog. https://blog.oup.com/2020/04/how-austerity-measures-hurt-the-covid-19-response/(accessed 28 April 2020).

[bibr43-03091325211065118] Folbre N (1986) Hearts and spades: paradigms of household economics. World Development 14(2): 245–255.

[bibr44-03091325211065118] Frade C Coelho L (2015) Surviving the crisis and austerity: the coping strategies of Portuguese households. Indiana Journal of Global Legal Studies 22(2): 631–664.

[bibr45-03091325211065118] Fregonese S (2017) English: lingua franca or disenfranchising? Fennia-International Journal of Geography 195(2): 194–196.

[bibr46-03091325211065118] Gayle R (2020) Creative futures of Black (British) feminism in austerity and Brexit times. Transactions of the Institute of British Geographers 45: 525–528. DOI: 10.1111/tran.12381.

[bibr47-03091325211065118] Geiger S (1990) What’s so feminist about women’s oral history? Journal of Women’s History 2: 169–182.

[bibr48-03091325211065118] Gillespie T Hardy K (2020) All in This Together? How a Decade of Austerity Cleared the Way for Covid-19 in Deprived Urban Areas. Global Development Institute. http://blog.gdi.manchester.ac.uk/decade-of-austerity-cleared-the-way-for-covid-19-in-deprived-urban-areas/

[bibr49-03091325211065118] Gopinath G (2020) ‘The Great Lockdown: Worst Economic Downturn Since the Great Depression’. IMF Blog. https://blogs.imf.org/2020/04/14/the-great-lockdown-worst-economic-downturn-since-the-great-depression/

[bibr50-03091325211065118] GOV.UK (2021) ‘NHS Workforce’. Ethnicity Facts and Figures. www.ethnicity-facts-figures.service.gov.uk/workforce-and-business/workforce-diversity/nhs-workforce/latest

[bibr51-03091325211065118] Gray M Smith S (2017) The Great Austerity Debate. www.geog.cam.ac.uk/research/projects/greatausteritydebate/

[bibr52-03091325211065118] Greer Murphy A (2017) Austerity in the United Kingdom: the intersections of spatial and gendered inequalities. Area 49(1): 122–124.

[bibr53-03091325211065118] Günel G. Varma C. Watanabe C. (2020) ‘A Manifesto for Patchwork Ethnography’, Member Voices. Fieldsights. https://culanth.org/fieldsights/a-manifesto-for-patchwork-ethnography (accessed 2 February 2021).

[bibr168-03091325211065118] Hall SM (2014) Ethics of Ethnography with Families: A Geographical Perspective. Environment & Planning A 46(9): 2175–2194.

[bibr169-03091325211065118] Hall SM (2016) Everyday Family Experiences of the Financial Crisis: getting By in the Recent Economic Recession. Journal of Economic Geography 16(2): 305–330.

[bibr170-03091325211065118] Hall SM (2017) Personal, relational and intimate geographies of austerity: ethical and empirical considerations. Area 49(3): 303–310.

[bibr171-03091325211065118] Hall SM (2019a) Everyday Life in Austerity: Family, Friends and Intimate Relations. Basingstoke: Palgrave-Macmillan.

[bibr172-03091325211065118] Hall SM (2019b) Everyday Austerity: Towards Relational Geographies of Family, Friendship and Intimacy. Progress in Human Geography 43(5): 769–789.

[bibr173-03091325211065118] Hall SM (2020a) The Personal is Political: Feminist Geographies of/in Austerity. Geoforum 110: 242–251.

[bibr174-03091325211065118] Hall SM (2020b) "It died once at playgroup, I didn’t know what to do": towards vital, vibrant material geographies of the mobile phone in austerity. *Social & Cultural Geography* *.* doi: 10.1080/14649365.2020.1843698

[bibr175-03091325211065118] Hall SM (2021) Reproduction, the life-course and vital conjunctures in austerity, UK. Medical Anthropology. doi: 10.1080/01459740.2021.1951261. 34237222

[bibr176-03091325211065118] Hall SM McIntosh K Neitzert E , et al. (2017) Intersecting Inequalities: The Impact of Austerity on Black and Minority Ethnic Women in the UK. London: Runnymede & Women’s Budget Group.

[bibr177-03091325211065118] Hall SM Stringer C (2017) Everyday Austerity zine, https://everydayausterity.wordpress.com/zine/ (accessed 20 May 2020).

[bibr54-03091325211065118] Hanisch C (1970) The personal is political. In: Firestone S Koedt (eds), Notes from the Second Year. New York, pp. 76–78.

[bibr55-03091325211065118] Haraway D (1988) Situated knowledges: the science question in feminism and the privilege of partial perspective. Feminist Studies 14: 575–599.

[bibr56-03091325211065118] Harrison E (2013) Bouncing back? Recession, resilience and everyday lives. Critical Social Policy 33(1): 97–113.

[bibr57-03091325211065118] Henry C (2015) Hospital closures: the sociospatial restructuring of labor and health care. Annals of the Association of American Geographers 105(5): 1094–1110.

[bibr58-03091325211065118] Himmelweit S Santos C Sevilla A , et al. (2013) Sharing of resources within the family and the economics of household decision-making. Journal of Marriage and Family 75(3): 625–639.

[bibr59-03091325211065118] Hitchen E (2016) Living and feeling the austere. New Formations 87: 102–118.

[bibr60-03091325211065118] Hitchen E (2019) The affective life of austerity: uncanny atmospheres and paranoid temporalities. Social & Cultural Geography: 1–24.

[bibr61-03091325211065118] Holmes H (2019) Unpicking contemporary thrift: getting on and getting by in everyday life. The Sociological Review 67(1): 126–142.

[bibr62-03091325211065118] Hopkins P (2019) Social Geography I: intersectionality. Progress in Human Geography 43(5): 937–947.

[bibr63-03091325211065118] Hopkins P (2018) Feminist geographies and intersectionality. Gender, Place & Culture 25(4): 585–590.

[bibr64-03091325211065118] Hopkins P Pain R (2007) Geographies of age: thinking relationally. Area 39(3): 287–294.

[bibr65-03091325211065118] Horton J (2016) Anticipating service withdrawal: young people in spaces of neoliberalisation, austerity and economic crisis. Transactions of the Institute of British Geographers 41(4): 349–362.

[bibr66-03091325211065118] Horton J Kraftl P (2009) Small acts, kind words and “not too much fuss”: implicit activisms. Emotion, Space and Society 2(1): 14–23.

[bibr178-03091325211065118] Horton J Pimlott-Wilson H Hall SM (2021) Growing Up and Getting By: International Perspectives on Childhood and Youth in. Hard Times. Bristol: Policy Press.

[bibr67-03091325211065118] Hyams M (2004) Hearing girls’ silences: thoughts on the politics and practices of a feminist method of group discussion. Gender, Place & Culture 11(1): 105–119.

[bibr68-03091325211065118] Jensen T (2018) Parenting the Crisis: The Cultural Politics of Parent-Blame. Bristol: Policy Press.

[bibr69-03091325211065118] Jensen T Tyler I (2012) Austerity Parenting: new economies of parent-citizenship. Studies in the Maternal 4(2): 1–5.

[bibr70-03091325211065118] Johnson A (2020) Throwing our bodies against the white background of academia. Area 52(1): 89–96.

[bibr71-03091325211065118] Joshi S McCutcheon P Sweet E (2015) Visceral geographies of whiteness and invisible microaggressions. ACME: An International E-Journal for Critical Geographies 14(1): 298–323.

[bibr72-03091325211065118] Jupp E (2021) Care, austerity and citizenship: story-telling as protest in anti-austerity activism in the UK. In: Hall SM Pimlott-Wilson H Horton J (eds), Austerity Across Europe: Lived Experiences of Economic Crises. London: Routledge.

[bibr73-03091325211065118] Jupp E (2017) Home space, gender and activism: the visible and the invisible in austere times. Critical Social Policy 37(3): 348–366.

[bibr74-03091325211065118] Jupp E Bowlby S Franklin J , et al. (2019) The New Politics of Home: Housing, Gender and Care in Times of Crisis. Bristol: Policy Press.

[bibr77-03091325211065118] Kobayashi A Peake L (1994) Unnatural discourse. ‘Race’ and gender in geography. Gender, Place & Culture 1(2): 225–243.

[bibr78-03091325211065118] Krumer-Nevo M (2017) Poverty and the political: wresting the political out of and into social work theory, research and practice. European Journal of Social Work 20(6): 811–822.

[bibr79-03091325211065118] Lewis G (2013) Unsafe travel: experiencing intersectionality and feminist displacements. Signs: Journal of Women in Culture and Society 38(4): 869–892.

[bibr80-03091325211065118] Lewis S (2018) Cyborg uterine geography: complicating ‘care’ and social reproduction. Dialogues in Human Geography 8(3): 300–316.

[bibr81-03091325211065118] Leyva del Rio S (2021) Decommodifying Housing, Deprivatising the Home: A Study of the Emergence of Self-Managed Cooperative Housing in the City of Barcelona. Unpublished Thesis, University of London, Birkbeck College.

[bibr82-03091325211065118] Longhurst R (1994) The geography closest in - the Body… the politics of pregnability. Australian Geographical Studies 32(2): 214–223.

[bibr83-03091325211065118] Longhurst R Johnston L (2014) Bodies, gender, place and culture: 21 years on. Gender, Place & Culture 21(3): 267–278.

[bibr84-03091325211065118] Longhurst R Ho E Johnston L (2008) Using ‘the body’ as an ‘instrument of research’: kimch’i and pavlova. Area 40(2): 208–217.

[bibr85-03091325211065118] MacLeavy J (2011) A ‘new politics’ of austerity, workfare and gender? The UK coalition government’s welfare reform proposals. Cambridge Journal of Regions, Economy and Society 4: 355–367.

[bibr86-03091325211065118] MacLeavy J Roberts S Strauss K (2016) Feminist inclusions in economic geography: what difference does difference make? Environment and Planning A: Economy and Space 48(10): 2067–2071.

[bibr87-03091325211065118] Massey D (2004) Geographies of responsibility. Geografiska Annaler: Series B, Human Geography 86(1): 5–18.

[bibr88-03091325211065118] Massey D (2013) Vocabularies of the economy. Soundings 54(54): 9–22.

[bibr89-03091325211065118] McCall L. (2005) The complexity of intersectionality. Signs 30: 1771–1800.

[bibr90-03091325211065118] McDowell L (1991) The baby and the bath water: diversity, deconstruction and feminist theory in geography. Geoforum 22(2): 123–133.

[bibr91-03091325211065118] McDowell L (1992) Doing gender: feminism, feminists and research methods in human geography. Transactions of the Institute of British Geographers 17: 399–416.

[bibr92-03091325211065118] McDowell L (1993) Space, place and gender relations: part II. Identity, difference, feminist geometries and geographies. Progress in Human Geography 17(3): 305–318.

[bibr93-03091325211065118] McDowell L (2012) Post-crisis, post-Ford and post-gender? Youth identities in an era of austerity. Journal of Youth Studies 15(5): 573–590.

[bibr94-03091325211065118] McDowell L (2015) Roepke lecture in economic geography—the lives of others: body work, the production of difference, and labor geographies. Economic Geography 91(1): 1–23.

[bibr95-03091325211065118] McDowell L (2017) Youth, children and families in austere times: change, politics and a new gender contract. Area 49(3): 311–316.

[bibr96-03091325211065118] Mitchell K Marston SA Katz C (2003) Life’s work: an introduction, review and critique. Antipode 35(3): 415–442.

[bibr97-03091325211065118] Mollett S Faria C (2018) The spatialities of intersectional thinking: fashioning feminist geographic futures. Gender, Place & Culture 25(4): 565–577.

[bibr98-03091325211065118] Montgomerie J (2016) Austerity and the household: the politics of economic storytelling. British Politics 11(4): 418–437.

[bibr99-03091325211065118] Montgomerie J Tepe-Belfrage D (2016) A Feminist moral-political economy of uneven reform in austerity Britain: fostering financial and parental literacy. Globalizations 13(6): 890–905.

[bibr100-03091325211065118] Morgan DHJ (1989) Strategies and sociologists: a comment on Crow. Sociology 23(1): 25–29.

[bibr101-03091325211065118] Müller M (2021) Worlding geography: from linguistic privilege to decolonial anywheres. Progress in Human Geography 45: 1440–1466.

[bibr102-03091325211065118] Mullings B (2009) Neoliberalization, social reproduction and the limits to labour in Jamaica. Singapore Journal of Tropical Geography 30(2): 174–188.

[bibr103-03091325211065118] Mullings B Peake L Parizeau K (2016) Cultivating an ethic of wellness in Geography. The Canadian Geographer 60(2): 161–167.

[bibr104-03091325211065118] Nast HJ (1994) Women in the field: critical feminist methodologies and theoretical perspectives. Professional Geographer 46(1): 54–66.

[bibr105-03091325211065118] Nowicka M (2020) Uni-Culti” myths and liberal dreams: brexit and austerity from the perspective of migrants. In: Contested Britain: Brexit, Austerity and Agency. Bristol, UK; Chicago, IL, USA: Bristol University Press, pp. 145–158.

[bibr106-03091325211065118] Oakley A (1981) Interviewing women: a contradiction in terms. In: Roberts H (ed), Doing Feminist Research. London: Routledge, pp. 30–61.

[bibr107-03091325211065118] Ortega M (2006) Being lovingly, knowingly ignorant: white feminism and women of color. Hypatia 21(3): 56–74.

[bibr108-03091325211065118] Oswin N Olund E (2010) Governing intimacy. Environment and Planning D: Society and Space 28(1): 60–67.

[bibr109-03091325211065118] Parizeau K Shillington L Hawkins R , et al. (2016) Breaking the silence: a feminist call to action. The Canadian Geographer/Le Géographe Canadien 60(2): 192–204.

[bibr110-03091325211065118] Pascoe Leahy C (2021) The afterlife of interviews: explicit ethics and subtle ethics in sensitive or distressing qualitative research. Qualitative Research 1–18. doi: 10.1177/14687941211012924.

[bibr111-03091325211065118] Pearson R (2019) A feminist analysis of neoliberalism and austerity policies in the UK. Soundings 71: 28–39. DOI: 10.3898/SOUN.71.02.2019.

[bibr112-03091325211065118] Pearson R Elson D (2015) Transcending the impact of the financial crisis in the United Kingdom: towards Plan F- a feminist economic strategy. Feminist Review 109: 8–30.

[bibr113-03091325211065118] Perrons D (2015) Gender equality in times of inequality, crisis and austerity: towards gender-sensitive macroeconomic policies. In: Bettio F Sansonetti S (eds), Visions for Gender Equality. Luxembourg: European Commission, pp. 16–20. http://ec.europa.eu/justice/gender-equality/files/documents/vision_report_en.pdf.

[bibr114-03091325211065118] Phinney S (2020) Rethinking geographies of race and austerity urbanism. Geography Compass 14(3): e12480.

[bibr115-03091325211065118] Pimlott-Wilson H (2017) Individualising the future: the emotional geographies of neoliberal governance in young people’s aspirations. Area 49(3): 288–295.

[bibr116-03091325211065118] Pollard J (2013) Gendering capital: financial crisis, financialization and (an agenda for) economic geography. Progress in Human Geography 37(3): 403–423.

[bibr117-03091325211065118] Pottinger L (2020) Treading carefully through tomatoes: embodying a gentle methodological approach. Area 1–7.

[bibr118-03091325211065118] Pottinger L (2017) Planting the seeds of a quiet activism. Area 49(2): 215–222.

[bibr119-03091325211065118] Power A Hall E (2018) Placing care in times of austerity. Social & Cultural Geography 19(3): 303–313.

[bibr120-03091325211065118] Raghuram P (2019) Race and feminist care ethics: intersectionality as method. Gender, Place & Culture 26(5): 613–637.

[bibr121-03091325211065118] Raynor R (2017) Dramatising austerity: holding a story together (and why it falls apart...). cultural Geographies 24(2): 193–212.

[bibr122-03091325211065118] Raynor RI (2018) Changing the Question from ‘The End of Austerity?’ to ‘What Ends in Austerity? Antipode Online https://antipodeonline.org/2018/11/19/what-ends-in-austerity/.

[bibr123-03091325211065118] Richardson MJ (2015) Embodied intergenerationality: family position, place and masculinity. Gender, Place & Culture 22(2): 157–171.

[bibr124-03091325211065118] Roberts A (2016) Household debt and the financialization of social reproduction: theorizing the UK housing and hunger crises. Research in Political Economy 31: 135–164.

[bibr125-03091325211065118] Robinson K (2020) Everyday multiculturalism in the public library: taking knitting together seriously. Sociology 54(3): 556–572.

[bibr126-03091325211065118] Rodó-de-Zárate M Baylina M (2018) Intersectionality in feminist geographies. Gender, Place & Culture 25(4): 547–553.

[bibr127-03091325211065118] Rose G (1993) Feminism & Geography: The Limits of Geographical Knowledge. Cambridge, UK: University of Minnesota Press.

[bibr128-03091325211065118] Rose G (1997) Situating knowledges: positionality, reflexivities and other tactics. Progress in Human Geography 21(3): 305–320.

[bibr129-03091325211065118] Rubery J Tarling R (1988) Women’s employment in declining Britain. In: Rubery J (ed) Women and Recession (Routledge Revivals), pp. 84–113.

[bibr130-03091325211065118] Sangster J (1994) Telling our stories: feminist debates and the use of oral history. Women’s History Review 3(1): 5–28.

[bibr131-03091325211065118] Schwiter K Strauss K England K (2018) At home with the boss: nigrant live-in caregivers, social reproduction and constrained agency in the UK, Canada, Austria and Switzerland. Transactions of the Institute of British Geographers 43(3): 462–476.

[bibr132-03091325211065118] Silvey R (2003) Gender and mobility: critical ethnographies of migration in Indonesia. In: Blunt A Gruffudd P May J , et al. (eds), Cultural Geography in Practice. London: Arnold, pp. 91–102.

[bibr133-03091325211065118] Silvey R (2012) Gender, difference and contestation: economic geography through the lens of transnational migration. In: Barnes TJ Pack J Sheppard E (eds), The Wiley-Blackwell Companion to Economic Geography. Chichester: Wiley-Blackwell, pp. 420–430.

[bibr134-03091325211065118] Sircar S (2021) Emplacing intersectionality: autoethnographic reflections on intersectionality as geographic method. Gender, Place & Culture: 1–20.

[bibr135-03091325211065118] Sisters of Frida . (2012) ‘Economic austerity or justification for denying disabled women’s Independence?’ Sisters of Frieda 21. http://www.sisofrida.org/economic-austerity-or-justification-for-denying-disabled-womens-independence/.

[bibr136-03091325211065118] Sisters Uncut . (2019) A Decade of Austerity. http://www.sistersuncut.org/2019/12/02/a-decade-of-austerity/.

[bibr137-03091325211065118] Skeggs B (2004) Class, Self and Culture. London: Routledge.

[bibr138-03091325211065118] Skop E (2015) Conceptualizing scale in the science of broadening participation of underrepresented groups in higher education. The Professional Geographer 67(3): 427–437.

[bibr139-03091325211065118] Sou G (2021) Concealing researcher identity in fieldwork and on social media: sexuality and silencing research participants. Area 53(3): 473–480. doi: 10.1111/area.12736. Available at: 10.1111/area.12736.

[bibr140-03091325211065118] Stacey J (1988) Can there be a feminist ethnography? Women’s Studies International Forum 11(1): 21–27.

[bibr141-03091325211065118] Stacey J (1998) Brave New Families: Stories of Domestic Upheaval in Late-Twentieth-Century America. California: University of California Press.

[bibr142-03091325211065118] Stenning A (2018) Feeling the squeeze: Towards a psychosocial geography of austerity in low-to-middle income families. Geoforum 110: 200–210.

[bibr143-03091325211065118] Strauss K (2013) Unfree again: social reproduction, flexible labour markets and the resurgence of gang labour in the UK. Antipode 45(1): 180–197.

[bibr144-03091325211065118] Strong S (2021) Facing hunger, framing food banks, imaging austerity. Social & Cultural Geography 1–18. doi: 10.1080/14649365.2021.1921247. Available at: 10.1080/14649365.2021.1921247.

[bibr145-03091325211065118] Summers BT (2019) Black in Place: The Spatial Aesthetics of Race in a Post-Chocolate City. UNC Press.

[bibr146-03091325211065118] Tarrant A (2014) Negotiating multiple positionalities in the interview setting: researching across gender and generational boundaries. The Professional Geographer 66(3): 493–500.

[bibr147-03091325211065118] Tarrant A (2018) Care in an age of austerity: men’s care responsibilities in low-income families. Ethics & Social Welfare 12(1): 34–48.

[bibr148-03091325211065118] Taylor F (2020) Love in the Time of Precarity: Snapshots. Queen Mary University London.

[bibr149-03091325211065118] Teeple Hopkins C (2015) Introduction: feminist geographies of social reproduction and race. Women’s Studies International Forum 48: 135–140.

[bibr150-03091325211065118] Tepe-Belfrage D Wallin S (2016) Austerity and the hidden costs of recovery: inequality and insecurity in the UK households. British Politics 11(4): 389–395.

[bibr151-03091325211065118] Thomson R Hadfield L Kehily MJ , et al. (2010) Family fortunes: an intergenerational perspective on recession. Twenty-First Century Society 5: 149–157.

[bibr152-03091325211065118] Torres RM (2018) A crisis of rights and responsibility: feminist geopolitical perspectives on Latin American refugees and migrants. Gender, Place & Culture 25: 13–36. DOI: 10.1080/0966369X.2017.1414036.

[bibr179-03091325211065118] Twigg J (2006) The Body in Health and Social Care. Basingstoke: Palgrave Macmillan.

[bibr153-03091325211065118] Valentine G (2007) Theorizing and researching intersectionality: a challenge for feminist geography. The Professional Geographer 59(1): 10–21.

[bibr154-03091325211065118] Valentine G (2008) Living with difference: reflections on geographies of encounter. Progress in Human Geography 32(3): 323–337.

[bibr155-03091325211065118] Volger CM (1994) Money in the household. In: Anderson M Bechhofer F Gershuny J (eds), The Social and Political Economy of the Household. Oxford: Oxford University Press, pp. 225–262.

[bibr156-03091325211065118] Walby S (1986) Patriarchy at Work. Cambridge: Polity.

[bibr157-03091325211065118] Welfare Imaginaries Zine (2020) Welfare Imaginaries: A Zine about the Past, Present and Future of Welfare. https://www.yumpu.com/en/document/read/63403337/welfare-imaginaries-zine (accessed 20 May 2020).

[bibr158-03091325211065118] Werner M Strauss K Parker B , et al. (2017) Feminist political economy in geography: why now, what is different, and what for? Geoforum 79: 1–4.

[bibr159-03091325211065118] Wilkinson C (2016) ‘Babe, I like your lipstick’: rethinking researcher personality and appearance. Children’s Geographies 14(1): 115–123.

[bibr160-03091325211065118] Wilkinson E Ortega‐Alcázar I (2019) The right to be weary? Endurance and exhaustion in austere times. Transactions of the Institute of British Geographers 44(1): 155–167.

[bibr161-03091325211065118] Wilkinson E Ortega-AlcÁzar I (2017) A home of one’s own? Housing welfare for ‘young adults’ in times of austerity. Critical Social Policy 37(3): 329–347.

[bibr162-03091325211065118] Winders J Smith BE (2019) Social reproduction and capitalist production: a genealogy of dominant imaginaries. Progress in Human Geography 43(5): 871–889.

[bibr163-03091325211065118] Windsong EA (2018) Incorporating intersectionality into research design: an example using qualitative interviews. International Journal of Social Research Methodology 21(2): 135–147.

[bibr164-03091325211065118] WBG . (2018) Plan A had failed. It is time Plan B. Women’s Budget Group UK Policy Briefings. Available at https://wbg.org.uk/analysis/plan-f-a-feminist-economic-strategy-for-a-caring-and-sustainable-economy/.

[bibr165-03091325211065118] WBG . (2020) Crises collide: women and COVID-19. Women’s Budget Group UK Policy Briefings. Available at https://wbg.org.uk/analysis/reports/crises-collide-women-and-covid-19/.

[bibr166-03091325211065118] Yuval-Davis N (2006) Intersectionality and feminist politics. European Journal of Women’s Studies 13(3): 193–209.

